# Impact of the COVID-19 Pandemic on Health, Well-being, and Quality of Work-Life Outcomes Among Direct Care Nursing Staff Working in Nursing Home Settings: Protocol for a Systematic Review

**DOI:** 10.2196/40390

**Published:** 2023-02-28

**Authors:** Trina Thorne, Yinfei Duan, Sydney Slubik, Carole A Estabrooks

**Affiliations:** 1 Faculty of Nursing University of Alberta Edmonton, AB Canada

**Keywords:** care aide, COVID-19 pandemic, direct care, frontline staff, mental health, nurse, nursing home, physical health, residential long-term care, work-life

## Abstract

**Background:**

Increased workload, lack of resources, fear of infection, and the suffering and loss of residents have placed a significant emotional burden on regulated and unregulated direct care nursing staff (eg, registered nurses, licensed practical nurses, and care aides) in nursing homes (residential long-term care homes). Psychological distress and burnout related to COVID-19 have been cited among direct care staff within nursing homes. Studies have also emphasized the resilience of direct care staff, who, despite the significant challenges created by the pandemic, remained committed to providing quality care. To date, only one nursing home–specific review has synthesized evidence from 15 studies conducted early in the pandemic, which reported anxiety, posttraumatic stress disorder, and depression among direct care staff.

**Objective:**

The objectives of this systematic review are to (1) synthesize all empirical evidence on the impact of the COVID-19 pandemic on direct care staffs’ mental health, physical health, and work-life outcomes; (2) identify specific risks and protective factors; and (3) examine the effect of strategies or interventions that have been developed to improve these outcomes.

**Methods:**

We will include all study designs reporting objective or subjective measurements of direct care staffs’ mental health, physical health, and quality of work-life in nursing home settings during the COVID-19 pandemic (January 2020 onward). We will search multiple databases (MEDLINE, CINAHL, Embase, Scopus, and PsycINFO) and gray literature sources with no language restrictions. Two authors will independently screen, assess data quality, and extract data for synthesis. Given the heterogeneity in research designs, we will use multiple data synthesis methods that are suitable for quantitative and qualitative studies.

**Results:**

As of December 2022, full text screening has been completed and data extraction is underway. The expected completion date is June 30, 2023.

**Conclusions:**

This systematic review will uncover gaps in current knowledge, increase our understanding of the disparate findings to date, identify risks and factors that protect against the sustained effects of the pandemic, and elucidate the feasibility and effects of interventions to support the mental health, physical health, and quality of work-life of frontline nursing staff. This study will inform future research exploring how the health care system can be more proactive in improving quality of work-life and supporting the health and psychological needs of frontline staff amid extreme stressors such as the pandemic and within the wider context of prepandemic conditions.

**Trial Registration:**

PROSPERO CRD42021248420; https://tinyurl.com/4djk7rpm

**International Registered Report Identifier (IRRID):**

DERR1-10.2196/40390

## Introduction

### Overview

The COVID-19 pandemic has exposed nursing home (residential long-term care home) residents and staff to a diverse set of challenges affecting their physical, psychological, and social well-being [1,2]. Evidence from the 2003 severe acute respiratory syndrome outbreak and emerging COVID-19 studies suggest that there will be global phenomena such as increased anxiety, depression, and trauma experienced by health care workers, which will likely have long-term impacts [3-9]. An analysis conducted in February 2021 of national data sets for 22 countries reported that nursing home deaths accounted for 41% of the total COVID-19 deaths (9%-64%) [10]. Concerted action to address the needs of staff working in nursing homes is needed to prevent and manage the impact of COVID-19 [11]. Therefore, a systematic review to synthesize all empirical evidence on the effects of COVID-19 on regulated and unregulated frontline nursing staff (registered nurses [RNs], licensed practical nurses [LPNs], and care aides) working in nursing homes is warranted.

### Background

Frontline nursing staff (RNs, LPNs, and care aides) working in nursing homes routinely experience challenges that are known to have negative effects on work-life. Prior to the COVID-19 pandemic, both regulated and unregulated direct care staff (RNs, LPNs, and care aides) experienced higher levels of burnout and mental health issues than the general population and difficulties meeting the daily care needs of residents [1,12-14]. Most nursing home residents have Alzheimer disease or another age-related dementia (~90% have cognitive impairment), multiple chronic diseases, recurrent medical problems, and complex emotional and social needs. The increasing complexity and dependency of resident populations, in the absence of commensurate resources, has led to unmanageable workloads for nursing home staff [12].

In the context of a COVID-19 outbreak, witnessing extreme suffering and the loss of residents with whom staff had long-term relationships has led to emotional exhaustion, which is a component of burnout [15]. Frontline nursing staff have also been subjected to substantial pressure from health authorities, who have introduced additional infection control and monitoring measures; from family members [16] who are concerned about residents’ well-being; and from the public and media. Despite a general acknowledgment that the challenges observed during the COVID-19 crisis in nursing home settings, particularly those that have contributed to severe resident outcomes, are symptoms of more systemic issues [1], the media has highly scrutinized nursing home staff. Additionally, nursing home staff have received little recognition for caring for persons infected with COVID-19 compared to hospital staff [16-18].

### Prior Work

Emerging evidence from nursing home settings across regions has generally reported higher rates of mental health symptoms, somatic symptoms, burnout, and reduced quality of work-life [15,19-21]. One study reported 71.3% (211/296) of the respondents had medium to high emotional exhaustion, and 64.2% (190/296) of the respondents had medium to high depersonalization [19]. The current and anticipated need for psychological or psychiatric support was 21.6% (64/296) and 41.0% (141/296), respectively [19]. Reports of job satisfaction vary. Two studies reported worsened job satisfaction [20,21], one of which compared nursing home staff data from COVID-19 to data from 2013 and noted an increase in burnout syndromes. In contrast, Blanco-Donoso and colleagues [15] found that 98.2% (329/335) of the participants agreed or totally agreed with the single item—*I feel satisfied to be able to help people*.

A descriptive study among Spanish nursing home staff reported 71% (149/210) of respondents had clinical stress, 29% (n=61) had clinical insomnia, and rates of clinical depression and anxiety were 49% (n=102) and 59% (n=124), respectively [16]. Riello and colleagues [22] found 44% (471/1071) of respondents passed the set threshold for moderate to severe anxiety and 44% (471/1071) of respondents passed the set threshold for moderate to severe symptoms of posttraumatic stress disorder (PTSD) [22]. A high risk of suicidality was found in 25% (10/40) of participants working in 2 nursing homes in Italy [23]. Previous COVID-19 infection was associated with a higher risk of suicidality [23].

Other studies examining individual and organizational risks and protective factors of mental health and work-life outcomes identified several modifiable and nonmodifiable characteristics. Factors influencing the rates and severity of mental health symptoms include access to personal protective equipment [15,22,24], professional support in the workplace [15,21,25], safety guidelines [24], psychological support [24], changes to work activities [25], increased workload [15,21], and direct contact with patients or colleagues who are COVID-19 positive [14,15,22]. Other factors increasing the risk of adverse outcomes include younger age [26], female sex [22,26], less education [22,26], and less work experience [26]. Shift workers, such as frontline nursing staff, have also been found to have higher rates of somatic symptoms [24]. Staff resilience has been associated with lower rates of anxiety, depression, insomnia, and stress [26].

Several reviews have been conducted during the COVID-19 pandemic in general health care worker populations. To date, however, only one review conducted by Gray and colleagues has synthesized a portion of the nursing home-specific evidence from 14 studies published between March 2020 and March 2021. This review reported a high prevalence of anxiety, PTSD, and depression among direct care staff in nursing home settings based on quantitative studies [27]. The synthesis of qualitative studies suggested various personal experiences among nursing home care staff, including psychological and mental health concerns, feeling undervalued and abandoned, fear of contagion, poor working conditions, lack of skills and knowledge, and support and the positive impacts of COVID-19 [27].

The number of studies on the impact of COVID-19 on nursing home staff has rapidly accumulated since the publication of Gray’s [27] review. An updated review is warranted to capture emerging studies and, most importantly, the enduring effects of COVID-19 as a sustained extreme stressor and potential effective interventions. This review is an important first step toward supporting the mental health of frontline staff during and after the COVID‐19 crisis and during other disease epidemics and pandemics. This is important for the health and well‐being of individual health care staff as well as nursing home residents.

### Objective

The purpose of this systematic review is to synthesize the empirical evidence on the effects of COVID-19 on frontline nursing staff who provide direct resident care, which includes RNs, LPNs, registered psychiatric nurses, and health care aides (HCAs), also called personal support workers (PSWs), direct care assistants, or nursing assistants, who worked in nursing homes during the COVID-19 pandemic. This systematic review will combine and synthesizes both quantitative and qualitative evidence. Its specific objectives are as follows: (1) to assess the impact of the COVID-19 pandemic on mental health (eg, stress, distress, depression, anxiety, PTSD, well-being, and somatic symptoms) and work-life outcomes (eg, burnout, job satisfaction, responsive behaviors directed toward staff, moral injury, distress, and working short-staffed) of frontline nursing staff (regulated and unregulated) working in nursing home settings (residential long-term care home); (2) to identify specific risks and protective factors; and (3) to examine the types and feasibility of strategies or interventions that have been developed to improve the outcomes of interest.

## Methods

### Protocol Registration

This protocol is reported following the Preferred Reporting Items for Systematic Reviews and Meta-Analyses Protocols (PRISMA-P) checklist (Multimedia Appendix 1) [28,29]. This protocol is registered with PROSPERO (CRD42021248420). Any amendments to this study protocol will be documented and filed with PROSPERO.

### Eligibility Criteria

All empirical study designs reporting the outcomes of interest for frontline nursing staff (regulated and unregulated) working in a nursing home setting and providing direct care during the COVID-19 pandemic will be considered for inclusion, without restriction on publication language. Articles published in languages other than English will be translated by authorized translators. We will exclude review articles; however, original studies included in reviews will be sourced and screened for inclusion. Articles that do not report primary study results, such as editorials, commentaries, opinion papers, theoretical papers or discussions, and anecdotal evidence will also be excluded.

### Population

#### Overview

Frontline nursing staff (regulated and unregulated) engage in direct contact with the resident (eg, physically providing care, administering medications, assisting with activities of daily living, providing social and emotional support, and assessing or developing care plans). In general, unregulated staff have a scope of employment defined by their job description rather than a scope of practice and are accountable to their employer. In contrast, regulated staff are governed by legislation, have legally defined scope of practice, and are answerable to an external nursing regulator that sets standards of practice and monitors the quality of care provided [30]. In contrast to synthesizing evidence on multiple workforce groups or settings, our review will investigate in detail the evidence available for frontline nursing staff to provide a comprehensive synthesis of a broader range of outcomes.

#### Inclusion Criteria

Frontline nursing staff include RNs, LPNs, registered psychiatric nurses, and HCAs, who are also called PSWs, direct care assistants, and nursing assistants. Studies may include other participant groups; however, they will limit data to only the subgroup of frontline nursing staff.

#### Exclusion Criteria

Nursing staff not providing direct care services (eg, management and administrative staff) will be excluded. Studies reporting on the outcomes of interest among frontline nursing staff exclusively from the perspective of a third-party informant, such as management (non–self-reported outcomes), will be excluded.

### Concept

#### Overview

We will study the effects of the COVID-19 pandemic as an extreme, sustained stressor on health and work-life outcomes among frontline nursing staff working in nursing homes. For this study, exposure to the COVID-19 pandemic is not limited to nursing homes with confirmed cases of COVID-19.

#### Inclusion Criteria

These criteria include studies reporting mental health, physical health, or work-life outcomes among frontline nursing staff during the COVID-19 pandemic (after January 2020).

#### Exclusion Criteria

These criteria include studies examining the impact of infectious diseases other than the current COVID-19 pandemic on health and work-life outcomes among frontline nursing staff.

### Context

#### Overview

According to the World Health Organization, nursing homes are responsible for the maintenance of well-being in older adults with a significant loss of functioning [31]. In this review, we define a nursing home (residential long-term care home) as a facility that provides living accommodations and 24-hour care services [32]. Direct care provided in nursing home settings ranges from health care services by regulated health care workers (eg, RNs and LPNs) to personal care provided by unregulated health care workers (eg, HCAs and PSWs) such as assisting with meals and bathing [32]. Studies will not be limited to settings with confirmed staff or resident cases of COVID-19.

#### Inclusion Criteria

These criteria include studies conducted after January 2020 in nursing home settings (eg, long-term care, residential care, and aged care) regardless of the country of origin. We will include studies in which there is a mix of different settings and will limit data to only the subgroup of nursing home staff when possible. If this is not possible, we will include the mixed data and explore how the inclusion affects the results by completing sensitivity analyses.

#### Exclusion Criteria

Studies conducted exclusively in nonnursing home settings will be excluded.

### Outcomes and Effects of Interest

#### Mental Health Outcomes

There is a wide range of mental health–related symptoms frontline nursing staff may experience [33]. Outcomes considered critical to this review include measures of mental health and well-being as these are anticipated to be highly impacted due to the conditions of COVID-19. Mental health describes an individual’s emotional and psychosocial well-being; it is defined by the World Health Organization as a state of well-being determined by interconnected social, psychosocial, and biological factors [34]. In this review, measures of mental health include but are not limited to experiences or symptoms of stress, distress, depression, anxiety, PTSD, and well-being.

#### Physical Health Outcomes

Physical health outcomes include somatic symptoms (eg, headache, insomnia, and emotional exhaustion). Since mental health is interrelated with somatic symptoms [34], all reported physical health outcomes and experiences will be included.

#### Work-Life Outcomes

Work-life outcomes include but are not limited to experiences or symptoms of burnout, job satisfaction, work engagement, responsive behaviors directed toward staff, moral distress, moral injury, and working short-staffed.

#### Risks and Protective Factors

We will extract all identified risks and protective factors affecting nursing home staff mental health and work-life outcomes. A risk factor is a condition, characteristic, or behavior that increases the risk of disease or injury [35]. Risk factors that can negatively impact mental health and work-life outcomes include increased workload, negative colleague and management relationships, lack of personal protective equipment, poor pandemic response planning, short staffing, staff turnover, extended work hours or mandatory overtime, and inability to take time off.

In contrast, protective factors are conditions, characteristics, or behaviors that improve well-being [35]. Resilience has been identified as an important protective factor that reflects the ability to cope with the negative effects of stress or adversity [1]. Additional examples of protective factors are an adequate supply of personal protective equipment, staff access to mental health resources, proper communication between staff and management, and paid sick days.

#### Type and Feasibility of Strategies or Interventions

Examples of interventions include workplace‐based psychological support strategies (peer support networks, employee wellness programs, psychological first aid, flexible schedules, regular information updates, and additional training), additional psychological support (guided and nonguided strategies, such as web-based therapy, web-based well-being and sleep apps, journaling, relaxation techniques, mediation and mindfulness programs, yoga, and coherent breathing), and interventions promoting or supporting healthy lifestyle and self‐care: eating, sleeping, exercising, following a routine, avoiding excess social media, staying in touch with family and friends, and doing things that are enjoyable [36,37].

### Search Strategy

A preliminary search of PubMed, Cochrane Library, Google Scholar (pages 1-10) and PROSPERO was conducted to ensure that no similar review is underway. The index terms and text words contained in the titles and abstracts of relevant papers were used to develop a full search strategy in consultation with a university health sciences librarian. We will conduct the search using combinations and synonyms of the core concept keywords for “COVID-19 pandemic,” “direct care staff,” “frontline staff,” and “nursing home.” We will search MEDLINE, CINAHL, Embase, Scopus, and PsycINFO databases, as well as sources of gray literature. These will include health discipline colleges and associations (eg, Registered Nurses Association of Ontario) and relevant conference programs (eg, Gerontological Society of America Annual Conference).

Additional sources of data will be identified by reviewing reference lists of relevant publications and reports, and contacting authors of relevant publications and reports to clarify published and seek unpublished information. The search strategy, including all identified keywords and index terms, will be adapted for each included database and information source. If reviews are identified, the citation list will be screened to ensure all relevant studies are included. To ensure all emerging intervention studies are included in our synthesis, we will repeat our search prior to publication. The results of the search will be reported in full and presented in a PRISMA flow diagram [28]. The search strategy is shown in Multimedia Appendix 2.

### Data Collection and Analysis

#### Identifying Relevant Literature

Screening will be undertaken using Covidence, a web-based citation software program, that facilitates web-based collaboration and blinding of reviewers during screening activities. All team members will receive training on the application of Covidence prior to screening. Two team members independently, using a 2-step process, will assess the results of the published and gray literature searches. In level 1, all records will be assessed against the following three screening questions: (1) Is the record a primary empirical study? (2) Is the study conducted in a nursing home (residential long-term care home) setting during the COVID-19 pandemic? (3) Does the study assess outcomes among frontline nursing staff that address the impact of the COVID-19 pandemic? All potentially relevant records, as well as those that do not contain enough information to determine eligibility, will be retained.

In level 2 screening, the full text of all retained records will be obtained and assessed for inclusion against the screening questions and inclusion criteria. Pilot testing, including the calculation of interrater agreement, will be completed at each stage of screening against the inclusion criteria for the review. All discrepancies will be resolved by consensus and, where necessary, by consulting a third senior team member. Reasons for exclusion will be documented. In situations where there are multiple reports (publications) for the same study that do not report additional outcomes or results, we will include the initial publication.

#### Data Extraction

We have adapted data extraction templates that were successfully used in past systematic reviews by the research team [38-40]. Using a small sample of eligible studies from the preliminary search, our study team worked collaboratively to establish and pilot-test the data extraction tool used for the review (Multimedia Appendix 3). The study team convened to compare the data extraction results from 2 reviewers and discuss the inconsistency that might stem from unclarity in the data extraction instructions and issues associated with the application of the tool. The discussions led to further modification and finalization of the tool. Google Forms will be used for the data extraction process so that reviewers can assess independently, and the results can be easily exported and compared. 

#### Methodological Quality

Two reviewers will independently assess the quality of each included study using the Joanna Briggs Institute Quality Appraisal Forms (Checklist for Randomized Controlled Trials, Checklist for Quasi-Experimental Studies, Checklist for Cohort Studies, Checklist for Case-Control Studies, Checklist for Case Series, Checklist for Prevalence Studies, Checklist for Analytical Cross-Sectional Studies, Checklist for Qualitative Research) [41] and the Mixed Methods Appraisal Tool, version 2018 [42]. Disagreements will be resolved through consensus and, if necessary, by consulting a third senior team member. For intervention studies, the research team will investigate if protocols have been published. When available, the protocols will be compared to the final publication to assess the presence of reporting bias. Appraisal results will be summarized narratively. Given the emergent nature of the topic and the descriptive focus of this review, we will not exclude studies based on quality.

#### Data Synthesis

We will use a parallel-results convergent design to synthesize both quantitative and qualitative evidence. According to the typology of synthesis designs for synthesizing qualitative and quantitative evidence developed by Hong and colleagues, a parallel-results convergent design analyzes and presents qualitative and quantitative evidence separately. The integration occurs during the interpretation of results in the Discussion section [43]. Data synthesis will be conducted separately for each objective. Quantitative studies will be stratified by outcome of interest or intervention and analyzed accordingly. If 3 or more studies investigate the same outcome using comparable methods, we will conduct statistical synthesis (meta-analysis). Details of the statistical models, methods and effect estimates, and measures of statistical heterogeneity will be reported. Where statistical pooling is not possible, findings will be presented in a narrative form, including tables and figures to aid in data presentation. For qualitative studies, careful comparisons will be made between the studies included in the synthesis to identify concepts and themes that are common or similar across the studies.

#### Mental Health, Physical Health, and Work-Life Outcomes

For the included quantitative studies, we will summarize and map outcomes of interest and the instruments used to measure the outcome (eg, established valid instruments and self-developed instruments). We will group the findings of included quantitative studies that reported the same outcome into main categories of outcomes and report the prevalence of the outcomes by presenting the original scores of instruments and cutoffs. Using narrative synthesis, we will report what is known about the impact of the COVID-19 pandemic on frontline nursing staffs’ work-life and mental health outcomes as well as how these outcomes have been examined. Narrative content analysis will be used to synthesize staffs’ experiences of mental health and work-life outcomes and generate themes (with representative quotes). This will allow us to identify associations between the COVID-19 pandemic and outcomes that may not have been quantitatively investigated. More details are provided in Multimedia Appendix 4.

#### Risks and Protective Factors

A narrative description of patterns derived from the findings will be used to understand the risks and protective factors that influence the experiences of frontline nursing staff. This will involve grouping studies by focus, methods, and outcomes to make comparisons between them. Synthesis will include calculating the proportion of quantitative studies that report significant results by factor (eg, the percent of studies that report peer support is a significant protective factor or that lack of peer support is a significant risk factor). The proportion of qualitative studies that describe significant factors (risk or protective) will also be reported.

#### Type and Feasibility of Strategies or Interventions

We anticipate a limited number of randomized controlled trials in this field and heterogeneity in study designs, interventions, and outcome measures. Accordingly, a quantitative synthesis of intervention effects may not be possible. In this regard, we will narratively synthesize the types of interventions developed or tested (psychological interventions and individual, group, or organizational-level intervention), the phase of the study (eg, feasibility study, pilot study, efficacy trial, and effectiveness trial), study design, and study findings.

#### Subgroups and Sensitivity Considerations

Where 3 or more studies investigate the same outcome using comparable methods, we will conduct subgroup analyses to determine whether results differ by (1) quality rating, (2) settings included in the study (multisetting vs nursing home only), and (3) the use of different measures of the same outcome. Gray et al [27] found evidence of worse outcomes among females and nurses when compared to other workforce groups [12,22,44]. If results indicate that other subgroups (eg, socioeconomically disadvantaged persons, racial or ethnic minorities, and unregulated staff) are disproportionately affected and there is an adequate number of studies with similar methods and outcomes representing each subgroup, we will conduct additional subgroup analyses. Comparison across or within countries may also be possible as emerging studies include large representative samples from over 20 countries, with the largest proportions from the United States, Canada, Spain, and the United Kingdom.

### Review Quality

This review will be conducted in accordance with Cochrane’s principles for systematic reviews [45], with adaptations for observational and qualitative study designs, which are frequently used in studies of staff mental health and quality of work-life outcomes. We will follow the PRISMA statement [46], a checklist of 27 essential items for transparent reporting of systematic reviews (Multimedia Appendix 1). We will also use the AMSTAR-2 tool, a 16-item checklist (developed as a quality appraisal tool for systematic reviews) to ensure our review meets quality standards and to avert any possible deficiencies in the conduct and reporting of our review [47].

### Ethics Approval

Ethical approval is not required as this project does not involve primary data collection.

## Results

### Overview

This study was funded in 2021. As of December 2022, full-text screening is complete and data extraction is underway. The expected completion date is June 30, 2023. Our preliminary search identified approximately 10 studies published in 2020, over 40 studies in 2021, and approximately 15 studies published in the first 4 weeks of 2022 that meet our eligibility criteria. These are primarily observational studies; however, 4 intervention studies, including 1 randomized controlled trial conducted in nursing homes, were identified. Most studies sampled frontline staff from nursing homes only; however, a portion include staff from multiple settings (eg, hospital, home care, and intensive care).

### Dissemination

Findings will be disseminated through conference presentations and publication in a peer-reviewed journal. Additionally, we will produce a plain-language summary of the findings for informing users on what is known on this topic and recommendations for use in practice. Any amendments made to this protocol when conducting the study will be outlined in PROSPERO and reported in the final manuscript.

## Discussion

### Overview

To date, there have been numerous COVID-19 studies of the impact on frontline nursing home staff with varied methods and, in some cases, disparate findings—leaving knowledge users with uncertainty. Therefore, a robust evaluation is required to gain a broader understanding of the impact of the sustained effects of COVID-19 on the nursing home workforce. This systematic review will uncover gaps in current knowledge to inform future research exploring how nursing homes can be more proactive in supporting the health and psychological needs of frontline nursing staff amid extreme stressors such as the pandemic and within the wider context of prepandemic conditions.

### Summary

Findings from the preliminary search suggest that nursing home workers have worse mental health outcomes than staff working in other health care settings [6]. Studies comparing outcomes by service setting (eg, nursing home, hospital, and primary care) report higher rates in one or more of clinical depression, anxiety, stress, PTSD, and insomnia among nursing home staff [12,14,44,48]. One study found that nursing home staffs’ risk of extended sick leave (<3 weeks) was almost 2-fold higher, while groups working in other health care had no elevated risk of sickness absence due to COVID-19 [49]. For frontline staff working in nursing homes, preexisting stressors may partially explain the increased risk of developing negative mental and physical health symptoms due to pandemic-related work conditions [12,16,50].

We identified 4 heterogeneous intervention studies in nursing home settings [36,51-54]. The first study aimed to prove the feasibility and effectiveness of treating anxiety and depressive symptoms among nurses who were highly mobilized during the acute phase of COVID-19 using a web-based (remote) Eye Movement Desensitization and Reprocessing (EMDR) protocol [36]. The authors concluded that web-based EMDR was both feasible and effective [36]. However, this study did not include a control group and all participants had received prior EMDR treatments from the therapist who provided the study intervention. This limits the ability to draw conclusions on the effectiveness of the EMDR protocol [36]. The second study was a randomized control trial that examined the effectiveness of self-help plus in reducing anxiety and posttraumatic symptomatology [51]. No difference in self-reported anxiety or posttraumatic symptomatology was found postintervention [51]. The third study used a mixed methods approach to evaluate the effectiveness of a web-based education program delivering just-in-time learning and best practices to improve staff confidence and comfort level when working with residents who were at risk, confirmed, or suspected of having COVID-19 [52]. The results indicated that the intervention effectively delivered time-sensitive information and best practices to support nursing home staff [52]. Lastly, Lathren and colleagues [53] explored the feasibility and effects of self-compassion training for certified nursing assistants working in nursing homes (n=34). Self-compassion training was found to be feasible and beneficial for the stressors that certified nursing assistants experienced during the COVID-19 pandemic [53].

### Strengths and Limitations

While there is significant interest in this topic, a preliminary search of the literature indicates that there is limited research available specifically focused on nursing home settings. Our inclusion of all designs will ensure that we provide the best evidence possible on the effects of the COVID-19 pandemic on frontline nursing staffs’ mental health and work-life outcomes that are protective and those that increase risk, as well as the effects of interventions to limit negative outcomes.

Second, the heterogeneity between study designs, outcomes of interest, and how these are measured may limit our ability to statistically compare results. Therefore, our analysis will include descriptive analysis, which will synthesize all available and potentially actionable evidence. We will also conduct a subgroup analysis if sufficient data are available to compare results when different measures are used to capture the same outcomes.

A third potential challenge we may face is that an insufficient number of intervention studies will be captured based on the timing of this review. The search will be repeated before synthesis to ensure all available intervention studies are included. Finally, as with all reviews, misinterpretation of the original research or data extraction errors is possible. To minimize this risk, 2 team members will independently engage in these activities, and discrepancies will be discussed to reach consensus. Should we encounter unforeseen circumstances where we need to deviate from our protocol, we will document and report the change in the completed review.

### Conclusions

Systematic reviews have a central place in informing decision-making from the level of individual patient care to health care policy makers [45,54]. To better understand the impact of the COVID-19 pandemic on frontline nursing staff health and work-life outcomes, identify the risks and protective factors, and determine which interventions are feasible and effective, a comprehensive synthesis of all existing evidence is needed. To date, there have been numerous COVID-19 studies related to its impact on frontline nursing home staff with varied methods and disparate findings, leaving knowledge users with uncertainty. This systematic review will inform the interpretation of the disparate findings to date and uncover gaps in current knowledge; doing so will guide future research exploring how the health care system can be more proactive in improving quality of work-life and supporting the health and psychological needs of frontline staff amid extreme stressors such as the pandemic and within the wider context of prepandemic conditions. Future research may examine specific interventions and policies to support nursing home staffs’ well-being, ultimately promoting better work-life and optimizing nursing home staffs’ ability to provide optimal quality of care to residents.

## Appendix

Multimedia Appendix 1 PRISMA Checklist.

Multimedia Appendix 2 Exemplar Search Strategy.

Multimedia Appendix 3 Data Extraction Termplate.

Multimedia Appendix 4 Exemplar Measures of Mental Health and Work-life Outcomes.

## Abbreviations

EMDR: Eye Movement Desensitization and Reprocessing
HCA: health care aide
LPN: licensed practical nurse
PSW: personal support worker
PTSD: posttraumatic stress disorder
RN: registered nurse

## Data Availability

No data sets were generated or analyzed during the development of this protocol. We will include all data generated or analyzed during the systematic review in the published manuscript (and its Multimedia Appendices). CAE will have full access to all data in the study and takes responsibility for the integrity of the data and the accuracy of the data analysis and synthesis.
