# Supplementary material for: Impact of the COVID-19 Pandemic on Health, Well-being, and Quality of Work-Life Outcomes Among Direct Care Nursing Staff Working in Nursing Home Settings: Protocol for a Systematic Review
Source: JMIR Res Protoc. 2023 Feb 28;12:e40390. doi: 10.2196/40390 (PMC9976775; doi:10.2196/40390)
Supplement: Multimedia Appendix 2 [file resprot_v12i1e40390_app2.docx]

**Additional file 2: Exemplar search strategies**

**MEDLINE search strategy**

1. ((((exp Coronavirus/ or exp Coronavirus Infections/ or (D614G or coronavirus* or corona virus* or OC43 or NL63 or 229E or HKU1 or HCoV* or ncov* or covid* or sars-cov* or sarscov* or Sars-coronavirus* or Severe Acute Respiratory Syndrome Coronavirus*).mp.) and ((20191* or 202*).dp. or 20190101:20301231.(ep).)) not (SARS or SARS-CoV or MERS or MERS-CoV or Middle East respiratory syndrome or camel* or dromedar* or equine or coronary or coronal or covidence* or covidien or influenza virus or HIV or bovine or calves or TGEV or feline or porcine or BCoV or PED or PEDV or PDCoV or FIPV or FCoV or SADS-CoV or canine or CCov or zoonotic or avian influenza or H1N1 or H5N1 or H5N6 or IBV or murine corona*).mp.) or ((((pneumonia or covid* or coronavirus* or corona virus* or ncov* or 2019-ncov or sars*).mp. or exp pneumonia/) and Wuhan.mp.) or (2019-ncov or ncov19 or ncov-19 or 2019-novel CoV or sars-cov2 or sars-cov-2 or sarscov2 or sarscov-2 or Sars-coronavirus2 or Sars-coronavirus-2 or SARS-like coronavirus* or coronavirus-19 or covid19 or covid-19 or covid 2019 or ((novel or new or nouveau) adj2 (CoV on nCoV or covid or coronavirus* or corona virus or Pandemi*2)) or ((covid or covid19 or covid-19) and pandemic*2) or (coronavirus* and pneumonia)).mp. or COVID-19.rx,px,ox. or severe acute respiratory syndrome coronavirus 2.os.)) and 20191201:20301231.(dt).
2. exp Nursing Homes/ or exp Homes for the Aged/ or exp Rehabilitation Centers/ or exp Skilled Nursing Facilities/ or (nursing home* or extended care* or care home*).mp. or ((senior* or continuing care or disabled or old age or geriatric* or elder care* or rehabilitat* or long term care) adj2 (lodge* or facility* or home* or residence* or centre* or center*)).mp.
3. mental health/ or posttraumatic growth, psychological/ or resilience, psychological/
4. ((mental or psychological) adj3 (health or wellbeing or "well-being" or wellness)).mp.
5. depression/ or exp mental fatigue/ or exp self-injurious behavior/ or exp suicide/ or exp stress, psychological/ or exp burnout, psychological/ or exp occupational stress/
6. exp affect/ or exp anger/ or anxiety/ or emotional regulation/ or psychological distress/ or fear/ or exp guilt/ or hostility/ or sadness/
7. "attitude of health personnel"/ or attitude to death/ or attitude to health/ or pessimism/
8. Job Satisfaction/
9. exp substance-related disorders/ or alcoholism/ or binge drinking/ or exp opioid-related disorders/ or stress disorders, traumatic/ or psychological trauma/ or stress disorders, post-traumatic/ or stress disorders, traumatic, acute/
10. "Sleep Initiation and Maintenance Disorders"/
11. (depress* or fatigue or exhaust* or "self-harm" or suicid* or stress or distress or burnout or anger* or anxiet* or anxious* or fear* or guilt* or hostil* or sadness or attitude* or pessimis* or "work-life-balance" or "work* satisfaction" or alcoholi* or "drug-use*" or "traumatic stress disorder*" or PTSD or insomni*).mp.
12. "Quality of Life"/

13. ("quality of life" or QOL or "life satisfaction" or HRQOL).mp.
14. ("responsive behavio?r*" or "behavio?ral and psychological symptoms" or BPSD or "neuropsychiatric symptom*" or NPS).mp.
15. or/3-14
16. 1 and 2 and 15

**Scopus search strategy**

( ( ( TITLE-ABS-KEY ( coronavirus* OR "corona virus*" OR covid* OR "sars-cov*" OR sarscov* OR "Severe Acute Respiratory Syndrome" OR sars ) ) OR ( ( TITLE-ABS-KEY ( ( wuhan OR beijing OR shanghai ) AND virus ) OR TITLE-ABS-KEY ( ( wuhan OR beijing OR shanghai ) AND pandemic ) ) ) ) AND ( ( ( TITLE-ABS-KEY ( nursing PRE/1 home* ) OR TITLE-ABS-KEY ( nursing PRE/1 center* ) OR TITLE-ABS-KEY ( nursing PRE/1 centre* ) OR TITLE-ABS-KEY ( nursing PRE/1 facilit* ) ) ) OR ( ( TITLE-ABS-KEY ( extended PRE/1 facilit* ) OR TITLE-ABS-KEY ( "long term" PRE/1 facilit* ) OR TITLE-ABS-KEY ( intermediate PRE/1 facilit* ) OR TITLE-ABS-KEY ( skilled PRE/1 facilit* ) ) ) OR ( ( TITLE-ABS-KEY ( geriatric W/1 institution* ) OR TITLE-ABS-KEY ( geriatric W/1 home* ) OR TITLE-ABS-KEY ( geriatric W/1 facilit* ) OR TITLE-ABS-KEY ( geriatric W/1 unit* ) OR TITLE-ABS-KEY ( geriatric W/1 center* ) OR TITLE-ABS-KEY ( geriatric W/1 centre* ) ) ) OR ( ( TITLE-ABS-KEY ( veteran* W/1 institution* ) OR TITLE-ABS-KEY ( veteran* W/1 home* ) OR TITLE-ABS-KEY ( veteran* W/1 facilit* ) OR TITLE-ABS-KEY ( veteran* W/1 unit* ) OR TITLE-ABS-KEY ( veteran* W/1 center* ) OR TITLE-ABS-KEY ( veteran* W/1 centre* ) ) ) OR ( ( TITLE-ABS-KEY ( "old-age" W/1 institution* ) OR TITLE-ABS-KEY ( "old-age" W/1 home* ) OR TITLE-ABS-KEY ( "old-age" W/1 facilit* ) OR TITLE-ABS-KEY ( "old-age" W/1 unit* ) OR TITLE-ABS-KEY ( "old-age" W/1 center* ) OR TITLE-ABS-KEY ( "old-age" W/1 centre* ) ) ) OR ( TITLE-ABS-KEY ( "convalescen* home*" OR "assisted care facilit*" OR "continuing care" OR "residential care" OR "care home*" ) ) ) AND ( ( ( TITLE-ABS-KEY ( psychological W/2 health ) OR TITLE-ABS-KEY ( psychological W/2 wellbeing ) OR TITLE-ABS-KEY ( psychological W/2 "well-being" ) OR TITLE-ABS-KEY ( psychological W/2 wellness ) ) ) OR ( ( TITLE-ABS-KEY ( mental W/2 health ) OR TITLE-ABS-KEY ( mental W/2 wellbeing ) OR TITLE-ABS-KEY ( mental W/2 "well-being" ) OR TITLE-ABS-KEY ( mental W/2 wellness ) ) ) OR ( TITLE-ABS-KEY ( depress* OR fatigue OR exhaust* OR "self-harm" OR suicid* OR stress OR distress OR burnout OR anger* OR anxiet* OR anxious* OR fear* OR guilt* OR hostil* OR sadness OR attitude* OR pessimis* OR "work-life-balance" OR "work* satisfaction" OR alcoholi* OR "drug-use*" OR "traumatic stress disorder*" OR ptsd OR insomni* ) ) OR ( TITLE-ABS-KEY ( "quality of life" OR qol OR "life satisfaction" OR hrqol ) ) OR ( TITLE-ABS-KEY ( "responsive behavior*" OR "responsive behaviour*" OR "behavioral and psychological symptoms" OR "behavioural and psychological symptoms" OR bpsd OR "neuropsychiatric symptom*" OR nps ) ) ) ) OR ( ( ( TITLE-ABS-KEY ( coronavirus* OR "corona virus*" OR covid* OR "sars-cov*" OR sarscov* OR "Severe Acute Respiratory Syndrome" OR sars ) ) OR ( ( TITLE-ABS-KEY ( ( wuhan OR beijing OR shanghai ) AND virus ) OR TITLE-ABS-KEY ( ( wuhan OR beijing OR shanghai ) AND pandemic ) ) ) ) AND ( ( ( TITLE-ABS-KEY ( psychological W/2 health ) OR TITLE-ABS-KEY ( psychological W/2 wellbeing ) OR TITLE-ABS-KEY ( psychological W/2 "well-being" ) OR TITLE-ABS-KEY ( psychological W/2 wellness ) ) ) OR ( ( TITLE-ABS-KEY ( mental W/2 health ) OR TITLE-ABS-KEY ( mental W/2 wellbeing ) OR TITLE-ABS-KEY ( mental W/2 "well-being" ) OR TITLE-ABS-KEY ( mental W/2 wellness ) ) ) OR ( TITLE-ABS-KEY ( depress* OR fatigue OR exhaust* OR "self-harm" OR suicid* OR stress OR distress OR burnout OR anger* OR anxiet* OR anxious* OR fear* OR guilt* OR hostil* OR sadness OR attitude* OR pessimis* OR "work-life-balance" OR "work* satisfaction" OR alcoholi* OR "drug-use*" OR "traumatic stress disorder*" OR ptsd OR insomni* ) ) OR ( TITLE-ABS-KEY ( "quality of life" OR qol OR "life satisfaction" OR hrqol ) ) OR ( TITLE-ABS-KEY ( "responsive behavior*" OR "responsive behaviour*" OR "behavioral and psychological symptoms" OR "behavioural and psychological symptoms" OR bpsd OR "neuropsychiatric symptom*" OR nps ) ) ) AND ( TITLE-ABS-KEY ( "care aide*" OR "care attendant*" OR "care guide*" OR "certified nursing assistant*" OR cna* OR "direct care worker*" OR "direct care staff" OR "geriatric health aide*" OR "health care assistant*" OR "nursing attendant*" OR "nursing home aide*" OR "personal care attendant*" OR "personal care nurse*" OR "personal care assistant*" OR "personal care attendant*" OR "personal support worker*" OR "residential aide*" OR "health care assistant*" OR "institutional aide*" OR "nurses aide*" OR "nursing assistant*" OR "nurs* aide*" OR "formal caregiv*" ) ) )
