# Supplementary material for: Impact of the COVID-19 Pandemic on Health, Well-being, and Quality of Work-Life Outcomes Among Direct Care Nursing Staff Working in Nursing Home Settings: Protocol for a Systematic Review
Source: JMIR Res Protoc. 2023 Feb 28;12:e40390. doi: 10.2196/40390 (PMC9976775; doi:10.2196/40390)
Supplement: Multimedia Appendix 3 [file resprot_v12i1e40390_app3.docx]

**Additional file 3: Data extraction template**

| Reference # |  |
| --- | --- |
| Reviewer and Date |  |
| Full Citation |  |
| Origin/Country |  |
| Study objective/research question |  |
| Research Design |  |
| Sample size (total and by group/setting) |  |
| Setting(s) |  |
| Population demographics (age, sex - total and by group/setting) |  |
| Intervention(s) description |  |
| Recruitment method(s) |  |
| COVID-19 infection and outbreak status |  |
| Type of outcomes measured/phenomena  of interest |  |
| Measures used |  |
| Data collection process |  |
| Data Analysis Process |  |
| Results |  |
| Conclusions |  |
| Strengths and limitations (reported and observed) |  |
| Funding |  |
| Comments |  |
