# Supplementary material for: Impact of the COVID-19 Pandemic on Health, Well-being, and Quality of Work-Life Outcomes Among Direct Care Nursing Staff Working in Nursing Home Settings: Protocol for a Systematic Review
Source: JMIR Res Protoc. 2023 Feb 28;12:e40390. doi: 10.2196/40390 (PMC9976775; doi:10.2196/40390)
Supplement: Multimedia Appendix 4 [file resprot_v12i1e40390_app4.docx]

**Additional file 4:** Exemplar measures of mental health and work-life related outcomes

| **Mental Health Domains** | **Potential Measures** |
| --- | --- |
| **Anxiety** | Generalized Anxiety Disorder 2 or 7‐Item (GAD-2 or GAD‐7), Spielberger Trait Anxiety Inventory, Self‐Rating Anxiety Scale (SAS), State‐Trait Anxiety Inventory, Depression, Anxiety and Stress Scale–21 Items (DASS‐21), Kessler Psychological Distress Scale |
| **Depression** | Patient Health Questionnaire (PHQ-2 or PHQ‐9), Beck Depression Inventory, Center for Epidemiologic Studies Depression Scale (CES‐D), Depression, Anxiety and Stress Scale–21 Items (DASS‐21) |
| **Stress** | Perceived Stress Scale (PSS-10), Ardell Wellness Stress Test |
| **Post-Traumatic Stress Disorder (PTSD)** | Stanford Acute Stress Reaction (SASR), Vicarious Traumatization Questionnaire, Impact of Event Scale (IES, IES‐R), Davidson Trauma Scale, PTSD Checklist‐Civilian Version (PCL‐C) |
| **General Mental Health** | Symptom Checklist 90 Revised (SCL‐90‐R), General Health Questionnaire (GHQ‐12 or GHQ‐28), Short Form questionnaire–all versions (SF-8, -20, or ‐36) |
| **Physical Health Domains** | **Potential Measures** |
| **Insomnia** | Insomnia Severity Index (ISI), Spielman's Insomnia Symptom Questionnaire (SISQ), Pittsburgh Sleep Quality Index (PSQI) |
| **Emotional Exhaustion** | Maslach Burnout Inventory questionnaire (MBIQ) subscale or 2 items “I feel burned out from my work” and “I have become more callous toward people since I took this job,” |
| **Work-life outcomes** | **Potential Measures** |
| **Burnout** | Oldenburg Burnout Inventory (OLBI), Maslach Burnout Inventory questionnaire (MBIQ) |
| **Job Satisfaction** | The Michigan Organizational Assessment Questionnaire, Job Descriptive Index (JDI), Job Satisfaction Survey (JSS) |
| **Work Engagement** | Utrecht Work Engagement Scale (UWES-9 or UWES-17), Work Engagement Scale (WES-3) |
| **Moral Distress/injury** | Measure of Moral Distress for Healthcare Professionals (MMD-HP), Moral Distress Scale-Revised (MDS-R); Moral Injury Event Scale (MIES), Moral Injury Symptom Scale (MISS-HP) |
| **Quality of Work-life** | Professional Quality of Life (ProQOL 5 or ProQOL21), Brief Index of Affective Job Satisfaction (BIAFJS), Work-Related Quality of Life scale (WRQoL) |

*Note.* This list is not exhaustive. We will include all studies examining these domains regardless of the measure(s) used.
